# Effectiveness of strengthening social protection and security programs in alleviating poverty in rural areas through multi-sector partnerships

**DOI:** 10.1016/j.heliyon.2024.e40485

**Published:** 2024-11-20

**Authors:** Elly Kuntjorowati, Soetji Andari, Reza Amarta Prayoga, Husmiati Yusuf, Saraswati Soegiharto, Siti Fatimah, Andayani Listyawati, Lisa Yuniarti, Suryani, Fatwa Nurul Hakim

**Affiliations:** a Research Center for Social Welfare, Village and Connectivity, National Research and Innovation Agency, Kampus BRIN Gatot Subroto, Jakarta, 12710, Indonesia; b Sociology Department, Faculty of Social and Political Science, University of Indonesia, Pondok Cina, Beji, Depok, West Java, 16424, Indonesia

**Keywords:** Social protection and security program 1, Strengthening social protection and social security 2, Factors causing poverty 3, Poverty alleviation 4, Multi-sectoral partnership 5

## Abstract

The effectiveness of strengthening social protection and security has proven to alleviate poverty in rural communities in the Garut Regency area, West Java Province, Indonesia, due in particular to excellent multi-sectoral cooperation. The social protection and security programs that the Indonesian Government provides are mainly targeted at the poor, as recorded in the social welfare data (DTKS). Poor people who are not registered with the DTKS cannot obtain social assistance programs. Therefore, it is necessary to strengthen the program so that the people who should be able to receive it can receive it. This is further strengthened through multi-sectoral collaboration between stakeholders, the business sector, academics, communities, and social media. The results of interviews with respondents show that strengthening social protection and security programs through multi-sectoral cooperation is very effective in helping reduce poverty in rural communities in Garut Regency, West Java Province, Indonesia.

## 1 Introduction

Social protection and security programs are closely related to poverty alleviation. Poverty alleviation and realizing economic prosperity have long since been on the world agenda and one of the Sustainable Development Goals (SDGs). Eradicating poverty in all its forms and dimensions, including extreme poverty, is the greatest global challenge and an indispensable requirement for sustainable development. All countries, policymakers, and collaboration partners are required to implement the plan that aims to free humanity from the tyranny of poverty [1] and move toward prosperity. Prosperity is when a person can fulfill his/her daily needs, both physical and spiritual, with money. Every human being has a different level of well-being, meaning that a person's level of well-being is not the same as another.

The Indonesian Government has implemented various family welfare programs throughout the years, including programs to consume nutritious food, 12 years of compulsory education for children, programs to reduce maternal and infant mortality rates, programs to improve the family economy, and programs empowering women. All of these programs are a part of reaching the SDGs. After all, economic welfare is either income generated or given under the circumstances to improve family welfare [2]. Not much research discusses the effectiveness of social protection and security programs for poverty alleviation, nor do they discuss the implementation process and how partnerships are established to strengthen these programs. Social protection and social security programs are becoming increasingly important in framing the relationship between the government and the poor. Many argue that poverty is a consequence from the poor themselves, such as being too lazy to work, having no income, and having low education, resulting in the lack of services acquired, decent work, or working for insufficient wages or very low wages. In cases such as these, it is the state that is responsible for the poor and must establish poverty alleviation policies [3]. Poverty alleviation refers to various efforts and strategies aimed at reducing and overcoming poverty, especially in rural areas, through various initiatives and policies to improve the economic conditions and welfare of the poor [4]. Many countries have established social protection and social security systems for workers whose wages and health benefits are minimal and for poor families earning only $1 per day [5]. The role of social protection and security programs in low-income developing countries has increasingly become a matter of concern in the sustainable development agenda. Ironically, only a few research results show the impact of social protection and social security programs on the welfare of poor families. Indonesia is one of the countries that implements social protection and security programs to alleviate poverty in rural areas through multi-sectoral means [6]. Alleviating poverty through multi-sectoral methods is the most effective method because it utilizes various resources and funds in an integrated and mutually facilitating manner. The success of multi-sectoral collaboration is highly dependent on the implementation of policies to evaluate the effectiveness of social protection and social security programs in alleviating poverty in rural communities [7]. Poverty alleviation refers to the absence or near absence of people or households below the poverty line, internationally, in a particular context. Poverty alleviation is a very urgent and top-priority concern in various countries. The main goal of the SDGs by 2030 is to end poverty and hunger in all its forms [8].

Determining the poor families that would become the target recipients of social protection and security programs is very important. If program recipients are not accurately targeted, it will pose a significant challenge in effectively distributing social assistance for poverty alleviation. Poor families who should be entitled to social protection and security programs often do not receive these benefits because the data on social assistance recipients fails to record their information accurately [9]. One such program is the Social Protection and Security Program, a poverty alleviation program intended for poor families, which is recorded in the integrated social welfare data (DTKS). The program takes the form of conditional cash transfers for poor families with several conditions that must be met. The poor families able to receive this social assistance have school-age children from elementary school (SD) to high school (SMA) who must attend school and program assistants. Program monitors will track the child's school attendance, and if a child is found to be absent, the assistance will be temporarily suspended until the child resumes school attendance. The number of poor people in Indonesia from year to year, according to data from official institutions, namely the Central Statistics Agency (BPS), in September 2021 was 26.50 million people, down 1.04 million people compared to March 2021. The following is data from BPS regarding the yearly number of poor people in Indonesia.

From Table 1, it can be interpreted that the number of poor people in Indonesia fluctuates from year to year. In 2018, the number of poor people was 25.95 million, while in 2019, it decreased by 25.14 million. In 2020, it increased to 26,42 million people. In 2021, there was a sharp increase, as great as 27.54 million people, and in 2022, there was a decrease of 26.16 million people. In 2023, the number decreased to 4189 million people, and it further decreased in 2024 to 2340 million people [10]. The poverty rate, which continues to decrease from year to year, is closely related to the existence of the Social Protection and Social Security programs, which are intended for the poor. This decrease in poverty rates is also closely related to the existence of the Sustainable Development Goals (SDGs) Document of Sustainable Development, which states that governments around the world agree to eradicate poverty by 2030 [11]. The West Java province, one of the provinces in Indonesia, has a higher-than-average number of impoverished individuals. Within West Java, the Garut Regency is notable for its significant population of poor residents [12]. The poor population in Garut Regency in 2021 reached 2,585,607 people, and the poverty line in Garut Regency is below the poverty line set by the World Bank, namely Rp 32,000 per person per day [13]. The high level of poverty in Garut Regency is caused by a lack of equality factors that support the advancement of the economy, such as education and health. Infrastructure is also a factor that causes low equality in the Garut Regency [12]. The cause of rural poverty in Indonesia is the low wages of agricultural laborers. The low wages of agricultural laborers positively impact poverty in rural areas in Indonesia [14]. An important aspect that needs to be considered in dealing with poverty in rural areas is an autonomous social institutional approach. This strategy involves mobilizing and leveraging existing social resources within the community, driven by collective awareness across multiple sectors. Through multi-sectoral joint awareness in overcoming poverty, the root of the problem of poverty in rural areas can be addressed so that a cross-actor network can be created to implement the poverty reduction program launched by the government.

**Table 1:**
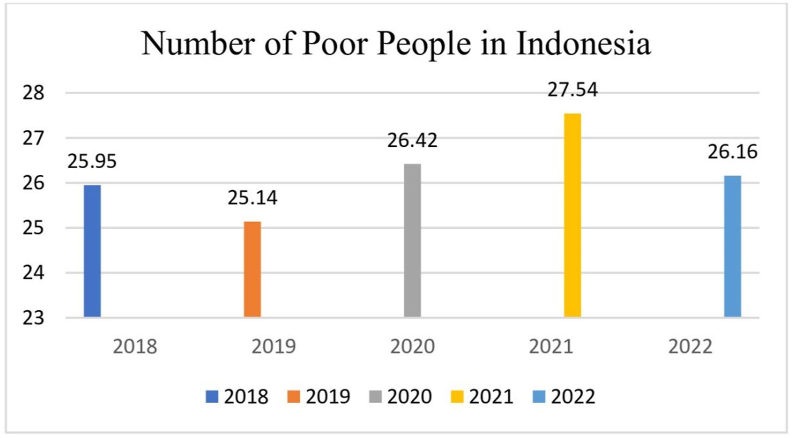
Number of poor people in Indonesia.

Central Statistics Agency, 2022 Central Statistics Agency. (2022). *Number of Poor People in Indonesia*. Central Statistics Agency Indonesia. https://www.bps.go.id/id/pressrelease/2023/01/16/2015/persentase-penduduk-miskin-september-2022-naik-menjadi-9-57-persen.html[Not Available in CrossRef][Not Available in Internal Pubmed][Not Available in External Pubmed].

The West Java Province, one of the provinces in Indonesia, has an above-average number of poor people. The Garut Regency is located in the West Java Province [15]. The number of poor people in Garut Regency back in 2021 reached 2,585,607 people, and the poverty line in Garut Regency was below the poverty line set by the World Bank, namely Rp. 32,000 per person per day [11]. The high poverty rate in Garut Regency is caused by a lack of equal distribution of supporting factors to advance the economy, such as education and health. Apart from that, infrastructure factors also cause low equality in the Garut Regency [12]. The factors of poverty alleviation in rural areas can be divided into six stages, and the essence of targeted poverty alleviation lies in helping those in need, identifying and assisting poverty-stricken households, accurately managing objects and actions, and assessing existing poverty levels and evaluating the effectiveness of poverty alleviation efforts [14].

An important aspect that needs to be considered when dealing with poverty in rural areas is the autonomous social institutional approach to encourage all social resources in society, which is driven by collective (multi-sectoral) awareness. Through joint (multi-sectoral) awareness in overcoming poverty, the root of the problem of poverty in rural areas can be addressed so that a cross-actor (multi-sectoral) network can be created when the government launches the poverty alleviation program. Poverty can stem from various sources, including natural, structural, or even cultural. Natural and economic poverty arises from limited natural, human, and other resources, reducing production opportunities and hindering development. Structural and social poverty, meanwhile, results from unequal development, inadequate institutional arrangements, and flawed development policies. On the other hand, cultural poverty is driven by attitudes or living habits considered inadequate, which can perpetuate a cycle of poverty.

The causes of poverty come from within and outside the poor population. Internal causes include the low quality of human resources and individual attitudes. Meanwhile, external causes are limited natural resources, social and institutional order in society, development policies, limited employment opportunities, and competition, which causes marginalization of the poor [16].

In developing countries, there are striking differences between rural and urban areas, especially in infrastructure development. Infrastructure development in Indonesia prioritizes development in urban areas rather than rural areas that are difficult to reach. These differences in development have a significant influence on poverty alleviation [17]. Social resilience of rural communities is used to describe a network of interconnected systems that directly impact society at the grassroots community level, including socio-economics, ecology, and the built environment. The characteristics of rural communities can be used as social innovation [17].

Multi-sectoral partnerships are essential in alleviating poverty, creating a challenge in realizing the SDGs. Partnerships at the local level are essential and influence the social system and the broader social environment, while global environmental impacts also influence the local level. The 2030 SDGs prioritize the importance of local partnerships in realizing the SDGs, especially in poverty alleviation. In this regard, this research wants to know the role of multi-sector partnerships in strengthening social protection and social security in alleviating poverty in rural communities [18].

### 1.1 Social protection program

The Indonesian Government, to overcome poverty and improve the social welfare of the poor residing in rural areas, has implemented social protection and social security programs, which have been strengthened through Social Welfare Law No. 11 of 2009. One particular program is targeted at poor families with an income of under $1 per day. The social protection program to alleviate poor families is called the Family Hope Program (PKH) . PKH is a social protection program for poor families that provides conditional social assistance. To receive this aid, families must meet specific requirements set by the program. Social protection programs are increasingly needed, especially in dealing with poverty problems. However, the success of program implementation still needs to be examined, especially to determine whether poverty alleviation programs such as PKH which provides direct cash assistance, can improve the welfare of poor families [19].

### 1.2 Family welfare program

Family social welfare programs aim to improve the quality of life of the community, especially the poor. Some of these programs include the Family Hope Program (PKH), a social assistance program for poor families. PKH is one of the Family Hope Program programs, a conditional social assistance program for Poor Families designated as beneficiary families (KPM), with the condition that they are registered in the integrated social welfare data (DTKS).To accelerate poverty alleviation, the Indonesian Government has implemented PKH since 2007. Social protection aid which is also known internationally as conditional cash transfers (CCT), has proven to be quite successful in overcoming poverty faced in these countries, especially the problem of chronic poverty. As a conditional social assistance program, PKH opens access for poor families, especially pregnant women and children, to utilize various health facilities and educational service facilities available around them. PKH benefits have also begun to be encouraged to include people with disabilities and the elderly while maintaining their level of social welfare [20].

### 1.3 Social security program

The social security program is a social protection program for all Indonesians from the Indonesian Government. This social security program in Indonesia is known as the Health Social Security Administering Body (BPJS). This program aims to ensure the fulfilment of community health needs. The social security program is a social insurance tied to a person's or worker's income. The program entails an obligation to pay a monthly fee [20]. However, poor families who receive the PKH program receive assistance from the Government called BPJS Contribution Assistance Recipients (PBI BPJS) to pay their monthly fee. Social security has a moderate role in narrowing the income gap, while social security contributions, especially health insurance contributions, have widened the income gap. Second, the impact of social security on rural residents and migrant workers is increasing. Third, social security effectively offsets the growing income gap due to market factors. Fifth, compared with high-income countries, the impact of social security systems operates on a much smaller scale [21].

### 1.4 Strengthening social protection and social security

The World Health Organization (WHO) defines health as maximally strengthening social protection for the physical, psychological, social, spiritual, economic, and fulfilling a person's basic needs [22]. Human resources and the social environment are vital to strengthening social protection and security [23]. Distribution of social assistance and social security programs that are equitable and well-targeted is an important aspect that can strengthen social protection and security programs. Such as the research results of Sui Yang et al. in Eastern China which show that equal social protection and social security distribution can overcome economic disparities due to market factors in Eastern China.

### 1.5 Factors causing poverty

All countries in the world face the issue of poverty and sustainable development. The SDGs stipulate that eradicating poverty is the top priority in sustainable development. Poverty alleviation requires the main contributors needed to find effective poverty alleviation policies. China has succeeded in finding the main factors causing poverty, namely the large number of young people who move from villages to cities because cities are more promising for their future, a side-effect of development strategies that often overlook the needs of the poor [24]. The level of poverty massively influences the economic growth of a region. If the poverty rate in a certain region is high, it will be increasingly difficult to eradicate poverty. Several factors influencing poverty levels include geographic location, education level, and population growth in the region [25]. Living in rural areas in the outermost regions of Indonesia in the era of globalization is very risky because they are left behind in the fields of education and technology. As a result, many young people are migrating to cities for better opportunities [26].

### 1.6 Poverty alleviation

Government and multi-sector policies in poverty alleviation is a coordinated effort between stakeholders, the business world, academics, social media, and community groups to reduce poverty and improve community welfare. The right innovation strategy is to appropriately manage the target recipients of social protection and social security programs so that the assistance truly benefits the poor. In line with the United Nations (UN) poverty eradication goal by 2030, poverty eradication must focus on rural communities, especially in strengthening social protection and security systems. Multi-sector partner-based poverty alleviation involves cross-sectoral coordination in fighting poverty.

Poverty means a lack of clothing, food, shelter, economy, health, and education, which prevents individuals from accessing quality education and healthcare and limits their ability to save money [27]. Poverty alleviation refers to the absence or near absence of people or households below the international poverty line in a particular context [28]. Poverty alleviation is a crucial point of the SDGs and therefore, countries must prioritize reducing income disparities while ensuring people have access to education, health, and nutrition services [29]. The poverty alleviation strategy is the development of urban and rural businesses, which aim to increase household income and women's ability to become entrepreneurs independently or with partners [4]. In dealing with poverty, the Indonesian Government attempts financial inclusion by analyzing data from the Central Statistics Agency to measure poverty. Measurements include fuel, lighting, education, employment, and access to banking and insurance [30]. The Indonesian Government's main priority in alleviating poverty is to improve the economy of poor households through social protection in the form of direct cash social assistance for poor families [13]. The Indonesian Government has established a poverty alleviation program through social protection and social security programs. Poverty alleviation necessitates multi-sectoral cooperation involving local governments, businesses, academics, mass media, and society. The first principle of poverty alleviation is social protection. The second principle is education, health, nutrition, and clean water services. The third principle is community empowerment, and the fourth is inclusive financial development, namely financial development for poverty alleviation, such as PKH and BPJS [31].

### 1.7 Characteristics of rural communities

Farmers are the main actors in rural communities, as observed from both geographical and participatory aspects [32]. Rural communities have distinctive characteristics: (1) High level of cooperation between residents (2) Simple living and very dependent on natural potential. (3) Limited access to public services and (4) infrastructure [33]. In developing countries in general, and in Indonesia in particular, there are differences in infrastructure development between rural and urban areas. The government tends to prioritize infrastructure development in urban areas over rural regions, where access is challenging and infrastructure development is most needed. This disparity directly affects the poverty dilemma, exacerbating rural communities' difficulties [17]. The striking differences between rural and urban areas have caused many rural young people to move to cities to more easily find work, resulting in a transformation from agriculture to industry in the city. These differences in conditions also cause poverty in rural areas. The characteristics of poor families are usually described by low human resources, weak natural and environmental resources, and a lack of inclusive financial development in poor areas [34].

### 1.8 Multi-sectoral partnerships

Multi-sector partnerships consists of five primary forms of partnerships: multi-party, business, academic, multi-media, and community. The essence of multi-sectoral partnerships is the importance of multi-sectoral participation, especially in poverty alleviation [35]. Current global poverty alleviation policies prioritize multi-sectoral actors and joint innovation as an effective model for accelerating poverty reduction. The key focus areas for these partnerships include engaging stakeholders, the business sector, academics, mass media, and the broader community. Each participant plays a distinct role in this collaborative effort: stakeholders act as policymakers, businesses engage through Corporate Social Responsibility (CSR), academics contribute through social work, mass media advocate for justice for the poor, and program recipient communities work alongside program assistants to implement and benefit from the programs. If these five partnership focuses run well, they can help eradicate poverty [36]. Multi-sectoral partnerships are essential in alleviating poverty and one of the challenges when realizing the SDGs. Partnerships at the local level are critical and influential for social systems and the broader social environment, while global environmental impacts also affect the local level. The 2030 Sustainable Development Goals (SDGs) emphasize the importance of local partnerships. In this context, this research aims to explore the role of multi-sector partnerships in enhancing social protection and security as part of efforts to eradicate poverty in rural communities [37].

## 2 Problem

1. What is the effectiveness of strengthening multi-sectoral-based social protection and social security programs for poverty alleviation in rural communities?
2. What are the forms of strengthening social protection and social security programs?
3. What institutions act as multi-sector partners in strengthening social protection and social security programs?
4. Can the role of multi-sector partnerships eradicate poverty in rural communities?
5. What is a multi-sector model for strengthening social protection and social security programs in alleviating poverty in rural communities?

## 3 Research purposes

1. Determine the effectiveness of strengthening multi-sectoral-based social protection and social security programs for alleviating poverty in rural communities.
2. Know the forms of strengthening social protection and social security programs.
3. Knowledge of multi-sector institutions that play a role in strengthening social protection and social security programs.
4. Understand the role of multi-sector partnerships in alleviating poverty in rural communities.
5. Understand the multi-sector model for strengthening social protection and social security programs to alleviate poverty in rural communities.

## 4 Research methods

Qualitative research methods are used in objective conditions and are the opposite of experimental research. In qualitative research, the researcher is the key to sampling, which uses purposive sampling [38]. In his work, Sugiyono stated that qualitative research places more emphasis on meaning than generalization [39]. Within the framework of research thinking, social reality is seen as a whole; therefore, suggestions are needed for further program improvements. Various disciplines have widely used qualitative research, and subjectivity and reflexivity are widely used concepts, but few have discussed how researchers engage effectively with essential aspects of research [40].

Data collection in this research used interview techniques and questionnaires aimed at multi-sector partnerships involved in strengthening social protection and social security in alleviating poverty in rural communities, including local governments, the business sector, academics, universities, and mass media. The number of informants interviewed for this research was 30 people, and to further strengthen the data, a focus group discussion (FGD) was held, wherein the participants represented all multi-sector elements and beneficiaries of social protection and social security programs. The FGD was conducted after obtaining approval from the Regional Development Planning Agency (Bappeda) and was held in the agency's meeting room. After obtaining approval, all multi-sector parties present, including the head of Bappeda, the Secretary of Bappeda, the PKH Coordinator, the PKH Facilitator, Social Media, Social Services, Beneficiary Families (KPM), business world, community leaders, and academics, were invited by Bappeda to attend the FGD. Procedural qualitative analysis is still quite a difficult challenge. Qualitative analysis is generally used in human research to assess a specific set of criteria [41]. The data analysis used in this research uses the context, input, process, and product evaluation (CIPP) model, where the data collection technique uses a questionnaire distributed to multi-sectors and beneficiaries of social protection and social security programs. In addition, this re [19]search employs focus group discussions (FGD), observation, and documentation [42].

An evaluative analysis is conducted first to assess the objectives of strengthening social protection and social security programs and the support provided. The next stage is multi-sector identification, program budget, implementation planning, time, and human resources. The third stage evaluates whether the ongoing process is good or requires changes. The final stage involves evaluating the impact of strengthening social protection and social security for beneficiaries [43].

## 5 Results

The results of the research on the Effectiveness of Strengthening Social Protection and Security Programs in Alleviating Poverty in Rural Areas Through Multi-sector Partnerships can be observed from the number of poor people in Garut Regency, the characteristics of the respondents, and the context of strengthening social and social protection. This context includes security, input for strengthening monitoring of social protection and social security, service processes for social protection and security programs, and effectiveness of social protection and security program products. In Indonesia, income disparities, especially among groups of the poor, are still a significant problem. Income inequality can also be interpreted as the gap between the rich and the poor. Inequality in income distribution and efforts to address it are central to development challenges and are primary objectives for policy development in many countries. It is crucial to include equal income distribution in the government's agenda. The Indonesian Government must implement policies to overcome income inequality. In this case, the government intervenes in previous policies to reduce inequality in Indonesia. Income inequality is a difference in income received or generated by society, resulting in unequal income distribution between communities. Research by Huang et al. [44] states that the relationship between economic development and income inequality can be positive or negative. In low-income developing countries, there is a negative relationship between income inequality and economic development. Developing countries have higher incomes than underdeveloped countries because of a positive relationship between inequality and economic development.

As of March 2023, the Gini ratio, which measures income inequality among the Indonesian population, was 0.388. In rural areas, the Gini ratio for the same period was 0.13. West Java's Gini ratio, which exceeds the national average, contributes to Garut Regency's ranking as the 9th (ninth) poorest area in the province, with a poverty rate of 10.42 %. The Cilawu District in Garut Regency, the focus of this research, has a significantly impoverished population of 48,288 individuals. Despite its proximity to economic and governmental centers in Garut Regency, Cilawu District experiences a relatively high poverty rate. Elementary and junior high school graduates dominate the characteristics of the poor community in Garut Regency, so it is tough to increase the Human Development Index (HDI), which is very useful for human resources in finding work and increasing income per person. This situation also contributes to a relatively high open unemployment rate in the Garut Regency, which is 7.6 %. Factors causing poverty in the Garut Regency include (1) Unequal economic development, (2) Income inequality, (3) Suboptimal government spending, (4) Low Development Index, and (5) Open unemployment [45]. From this data, the priority for economic development and equal distribution of income in Garut Regency is to reduce the poverty rate by minimizing exclusion errors in the Integrated Social Welfare Data (DTKS) so that there are no inaccuracies in targeting recipients of social assistance programs, social protection, and social security.

The number of poor people in Garut Regency in March 2023 was 260.48 thousand. The poverty line in March 2023 was recorded at IDR 367,681/capita/month, an increase from March 2022 of IDR 335,134/capita/month. The poor population in rural areas is 9.77 %. The number of extremely poor people in 2023 was 82,170 people. The number of open unemployed there in August 2023 was 7.6 %. The number of Garut Regency residents who have entered the Integrated Welfare Data (DTKS) is 185,803, while those who have not entered the DTKS are 64,130.

These groups of poor people who have not been registered in the DTKS need attention from the government and multi-sector partners. By doing so, they can access social assistance, protection, and security programs, similar to other impoverished individuals, helping to alleviate their housing cost burdens. The number of KPM recipients of the social protection program in the form of conditional cash social assistance is 126,759 people, and BLT food social assistance is 283,562. These two programs are social protection programs for poor families registered with DTKS. The social security program is an insurance program that requires monthly fees from its participants, but for poor people who are registered with the DTKS, the fees are borne by the government. Social security programs include health services through ownership of the Healthy Indonesia Card (KIS) and 12-year compulsory education services through the Smart Indonesia Card (KIP) ownership.

1 Respondents' Education Levels

One of the causes of poverty is low human resources, namely the level of education. The level of education has a direct effect on employment [46]. The following table will show the education level of multi-sector respondents, including those from the local government, the academic sector, the business sector, social media, and community groups.

Table 2 shows the various levels of education of the respondents. As many as 13.3 % of respondents have an elementary school education, they are beneficiary families of social protection and social security programs commonly referred to as KPM. Poverty causes their low level of education, therefore the Family Hope Program (PKH) which is a social protection program requires KPM children to attend school up to high school and even to college, this is to improve the future of children to be more prosperous. Those with master's degrees are stakeholders from the Garut Regency Bappeda officials who grant research permits at the location. As many as 50 % of respondents have bachelor's degrees and are workers in the business sector, academic sector, social media, social protection and security program coordinators, and program assistants. As many as 20 % of respondents with a high school education are active on social media.

2 Respondents' incomes

**Table 2:** Respondents' education.

**No**	**Education Level**	**F**	**%**
1	Elementary School	4	13,3
2	Junior High School	1	3,3
3	Senior High School	6	20
4	Bachelor Degree	15	50
5	Stratum Two	4	13,3
Amount		N = 30	100 %

Classical economists from World War I to World War II argue that a person's welfare can be determined by income. This opinion relies on a neo-classical combination of goods and services. According to this view, welfare is a combination of goods and services, including income and providers of income-covering material and non-material aspects such as health, education, and quality of life. Production is conceptualized as an indirect exchange, wherein people, by demanding goods, essentially demand the services of the factors of production [47]. The basic idea of economists is that economic development has the same meaning as economic growth and prosperity [48]. Income has a direct impact on food security and the overall social welfare of individuals. Income can also be developed to carry out productive economic efforts on a small or macro scale [49].

Table 3 shows that 16.7 % of respondents earn IDR1,500,000 per month, they are beneficiary families (KPM). Thanks to the assistance of social protection and social security programs, their income is above the poverty line set by the World Bank at US$1 per day, equivalent to IDR 28,900. Some KPM have even been able to open stalls, to increase their income, the social assistance they receive is used as business capital. Those who earn IDR5,000,000 are stakeholders and Bappeda officials. The informants of this study were regional officials of Garut Regency who are members of Bappeda. Those who earn IDR3,100,000 work as assistants in social protection and social security programs, while individuals with incomes of IDR4,100,000 act as program coordinators.

3 Gender of Respondents

**Table 3:** Respondent's income.

**No**	**Income**	**F**	**%**
1	1,500,000	5	16.7
2	1,800,000	1	3.3
3	2,000,000	1	3.3
4	3,000,000	2	6.7
5	3,100,000	7	23.3
6	4,000,000	4	13.3
7	4,100,000	5	16.7
8	4,500,000	1	3.3
9	5,000,000	4	13.3
Amount		N = 30	100 %

Men and women are differentiated based on body shape, character, workload, and reproductive function, often referred to as gender [50]. Someone who behaves femininely is called a woman, and someone who behaves masculinely is called a man. Puberty is when gender differences between men and women appear [51]. The following table shows the gender of respondents, which is closely related to social protection and social security programs.

From Table 4, it can be seen that the gender of the majority of respondents are female, making up 18 people or 60 %. Meanwhile, 12 out of the total 30 respondents, or 40 %, are male. The Social Protection and Security program mandates that recipients must be women from impoverished households. Eligibility is verified through the possession of a social welfare card. The social assistance component in social protection and security programs is closely related to the recipient's gender because program recipients must be women in poor households. These components, among other things, are intended for pregnant women, children under five, children's education from elementary to high school, and nutritional fulfillment for mothers and children. Forty percent of male informants are stakeholders in social protection and social security programs.

4 Interview Results Regarding the Goals of Strengthening Social Protection and Social Security Programs

**Table 4:** Gender of respondents.

**No**	**Gender**	**F**	**%**
1	Man	12	40 %
2	Female	18	60 %
Amount		N = 30	100 %

Based on the answers of the 30 respondents interviewed, they reported that there had been an expansion of social protection and social security programs in their area, allowing previously unassisted poor individuals to receive support. This expansion is part of broader poverty alleviation efforts. The respondents noted that the Ministry of Social Affairs is working on recording and incorporating data on impoverished individuals into the Integrated Social Welfare Data (DTKS). We also inquired about the role of multi-sectoral collaboration. According to the respondents, various sectors, including stakeholders, play a crucial role in registering impoverished individuals who have not registered in the DTKS. Corporate Social Responsibility (CSR) plays a role in providing educational assistance to poor people who have not yet registered within the DTKS, helping to pay health insurance contributions such as the health insurance administering body (BPJS), medical assistance, and free ambulance assistance. Academics are responsible for advocating the need to improve the data on impoverished communities in DTKS, as the current data remains disorganized. Social media platforms highlight the importance of ensuring justice for those who have not yet received social protection and social security programs. We also asked program recipient families about strengthening social protection and social security programs, and they said that strengthening these programs could be known through good public services.

5 Interview Results Regarding Inputs for Strengthening Social Protection and Social Security

Program services must reach all levels of the poor and vulnerable population, including people with disabilities, the elderly, and orphans, to strengthen social protection and social security. Strengthening social protection and security can include, among other things, strengthening social security institutions, such as expanding membership and coordinating with regional governments and multi-sector partners. The results of interviews regarding input into the family welfare program (PKH), a social protection program, include monitoring the attendance of children receiving the PKH program at school by PKH assistants because the program requires the child to meet 80 % of the total attendance. If the child is absent from school and the attendance is less than 80 %, the PKH social assistance can be temporarily suspended until the child enters school. PKH facilitators conduct monthly monitoring of program recipients in group settings. These sessions include empowerment activities and circulating information related to the PKH program. The interview results also indicated that during the PKH program, the health of pregnant women and toddlers is being monitored, as the PKH program specifically targets improving health outcomes for these groups. At monthly meetings PKH facilitators hold with program recipients, they also discuss their economic conditions before and after receiving the program and whether there have been changes towards improvement. Additionally, they review how the assistance is utilized, including its potential use as capital for small businesses. The accuracy of the target recipients of the social assistance program is also continuously monitored because there is a graduation procession each year where those who are no longer eligible can be replaced by those who are more suitable to receive.

6 Interview Results Regarding the Social Security and Protection Program Service Process

The Indonesian Government's poverty alleviation agenda goes through two stages. First, it includes social protection, social empowerment programs, and social security initiatives, all of which fall under the broader umbrella of social protection. Social protection can help tackle poverty by reducing inequalities and building household resilience in the face of unexpected events, such as illnesses or accidents, which can further push poor people into poverty. The results of their interviews stated that Garut Regency facilitates social services for poverty alleviation because Garut Regency has a program for its residents, especially those who do not or have not received the PKH program. This program is called the Community Hope House Integrated Service (Lapad Ruhama), which will answer poverty problems ranging from uninhabitable houses to economic problems. This initiative aims to bridge the economic gap that exists in the Garut Regency. Lapad Ruhama is a policy of the Garut Regency regional government to overcome poverty in its area. It serves as a solution, especially for poor residents who cannot get social assistance programs in the form of social protection and social security, because they are not recorded in the DTKS.

7 Interview Results Regarding the Effectiveness of Strengthening Social Protection and Social Security Programs

The Family Hope Program, abbreviated as PKH, is a social protection program for poor households that was initiated in 2007. The assistance that the program provides is conditional social assistance for poor families. The form social protection aid, known internationally as a conditional cash transfer (CCT), is quite effective in overcoming poverty in these countries. The results of interviews with respondents revealed that social protection and security programs were very effective in helping eradicate poverty because they were directly related to the economy of poor families. Socialization regarding program disbursement procedures is effective for helping poor families who receive the program. Opening a collective account book for prospective program recipients proves helpful and practical. According to the recipients, disbursing social assistance through designated bank agents is highly effective. The accuracy of targeting program recipients, the accuracy of the amount of social assistance, the accuracy of administration, and the timeliness of social assistance disbursement are very effective in alleviating poverty. Correct data collection on poor families in DTKS effectively alleviates poverty. The role of stakeholders, in this case, the Regional Government of Garut Regency, which provides separate social assistance in addition to the PKH program, is very effective for equal distribution of poor families who do not receive the PKH program. The PKH assistance for pregnant women who receive the PKH program is IDR. 3000,000 per year is very effective in helping the health of pregnant women. The PKH assistance for early childhood of IDR 3000,000 per year is very effective in helping children's growth and development. The aid for elementary school children who receive the program is IDR. 900,000,- per year is very effective for children's education. The PKH assistance for junior high school children amounting to IDR 1,500,000 is very practical for the continuity of children's education. The PKH assistance for high school children is IDR. 2,000,000 per year is very effective in helping children's education continue. Social assistance of IDR 2,400,000 for children with disabilities has proven very effective in supporting their needs. Similarly, the same amount of IDR 2,400,000 for elderly individuals helps improve the welfare of seniors in impoverished families. If a poor family receiving the program qualifies for multiple components, they can benefit from all applicable forms of assistance.

## 6 Discussion

From Tables 1 and it can be seen that the poverty rate in Indonesia from 2022, 2023, and 2024 continues to decline. This is inseparable from government policies, namely social protection and social security programs implemented by the Indonesian Government. This decline in poverty rates is also closely related to the Sustainable Development Goals (SDGs) Document from the United Nations (UN), which states that by 2030, governments around the world have agreed to eradicate poverty. Table 2 shows that poverty causes someone to be unable to pursue higher education because there are no funds to pursue higher education. PKH, as one of the social protection programs, requires children from an early age to adulthood to go to school. Children's attendance at school is monitored by program assistants, and if a child is found not to be in school, PKH assistance will be halted until the child returns to school. Table 3 shows that due to the assistance of social protection programs, KPM's income is already above the poverty line set by the World Bank at US$1 per day, or equivalent to IDR28,900. Some KPM recipients have even been able to develop the social assistance they receive by opening grocery stalls. They are greatly helped by this program so that it can lighten their burden, especially for children's education needs and in accessing health services for pregnant and lactating mothers and early children under six years.

## 7 Conclusion

From the interviews that have been analyzed from various angles, it can be concluded that strengthening social protection and insurance programs alleviates poverty effectively. In terms of strengthening social protection and security programs, respondents reported that there has been an expansion of these programs in their area. This expansion ensures that previously unassisted poor individuals can now access social assistance, contributing to poverty alleviation efforts. The results of the interviews regarding strengthening social protection and social insurance programs show that strengthening social protection and social security is expected to cover all levels of poor and vulnerable society, such as people with disabilities, the elderly, and orphans who must receive program services. Based on the results of interviews regarding the social protection and social security program service process, the results state that Garut Regency facilitates social services for poverty alleviation because Garut Regency has its program for its residents, especially those who do not or have not received the PKH program. This program is called the Community Hope House Integrated Service (Lapad Ruhama), which will answer poverty problems ranging from uninhabitable houses to economic problems. Its implementation aims to bridge the economic gap in the Garut Regency. Based on the results of interviews with respondents regarding the effectiveness of social protection and social security programs, social protection and security programs are very effective in helping to eradicate poverty because they are directly related to the economy of poor families. Socialization about program disbursement procedures has proven very effective in assisting poor families who benefit from the program. Collectively opening account books for prospective recipients is also seen as highly helpful and effective. Disbursing social assistance through designated bank agents has been reported as very effective.

Critical factors in alleviating poverty include the accuracy of targeting program recipients, the appropriateness of social assistance amounts, the precision of administrative processes, and the timeliness of assistance disbursements. Accurate data collection on poor families in the DTKS is crucial for effective poverty alleviation. Additionally, the involvement of stakeholders, particularly the Regional Government of Garut Regency, in providing separate social assistance alongside the PKH program significantly contributes to the equitable distribution of aid to poor families who do not receive PKH benefits.

Overall, it can be concluded from the interviews regarding the social protection and security program, the social protection and security program service process, and the resulting results all show that social protection and social security programs effectively alleviate poverty. Multi-sectors are involved in helping to alleviate poverty. Multi-sector partnerships create programs to deal with poor people who do not receive programs, which is done to overcome the poverty gap in their region.

Economic development that can provide equal distribution of income and social welfare for the entire population of Indonesia is called inclusive economic development. The most crucial thing in inclusive economic development is to emphasize appropriate policies to equalize the income of the poor [52]. Economic development is in line with income distribution, especially for the poor. The Indonesian Government's policy in equalizing income is through social protection and social security programs, which are programs for alleviating poverty and equalizing income. This program not only targets poor people but also groups vulnerable to poverty.

Strengthening social protection and social security through a multi-sectoral approach has proven highly effective in alleviating poverty in rural communities. Interview results confirm that these programs significantly impact the poverty alleviation effort, highlighting the need for continued implementation and support.

The implications of the results of this research can be emulated for other regions, especially multi-sectoral areas, to develop poverty alleviation programs in their regions for poor residents who have not yet been recorded in the national database. The results of the study show that the program beneficiaries can experience welfare, manage family finances well, and meet what they need, not what they want. In addition to guaranteed health services, they are kept safe from having to drop out of school, particularly pregnant and breastfeeding women, as well as toddlers and children.

## CRediT authorship contribution statement

**Elly Kuntjorowati:** Writing – original draft, Validation, Formal analysis. **Soetji Andari:** Writing – review & editing, Writing – original draft, Methodology, Conceptualization. **Reza Amarta Prayoga:** Writing – review & editing, Formal analysis, Conceptualization. **Husmiati Yusuf:** Formal analysis, Conceptualization. **Saraswati Soegiharto:** Writing – review & editing, Validation, Supervision. **Siti Fatimah:** Resources, Project administration, Data curation. **Andayani Listyawati:** Methodology, Formal analysis, Conceptualization. **Lisa Yuniarti:** Project administration, Methodology. **Suryani:** Validation, Methodology, Data curation. **Fatwa Nurul Hakim:** Formal analysis, Data curation, Conceptualization.

## Ethics statement

This research adhered to the ethical standards set forth by the National Research and Innovation Agency (No: 438/KE.01/SK/07/2023). Informed consent was obtained from all respondent and relevant parties at Garut Regency, West Java. The authors declare that they have no conflicts of interest.

## Data availability statement

This research data has been stored in the BRIN repository.

## Declaration of competing interest

The authors declare that they have no known competing financial interests or personal relationships that could have appeared to influence the work reported in this paper.
